# Cultural adaptation and validation of the “Kidney Disease and Quality of Life - Short Form (KDQOL-SF™) version 1.3” questionnaire in Egypt

**DOI:** 10.1186/1471-2369-13-170

**Published:** 2012-12-13

**Authors:** Samar Abd ElHafeez, Sunny A Sallam, Zahira M Gad, Carmine Zoccali, Claudia Torino, Giovanni Tripepi, Hala S ElWakil, Noha M Awad

**Affiliations:** 1 Epidemiology Department, High Institute of Public Health, Alexandria University, Alexandria, Egypt; 2 CNR-IBM, Epidemiology and Pathophysiology of Renal diseases and Hypertension of Reggio Calabria, Reggio Calabria, Italy; 3 Nephrology Department, Faculty of Medicine, Alexandria University, Alexandria, Egypt

**Keywords:** Chronic kidney disease, Egypt, Health-related quality of life, KDQOL-SF^TM^ 1.3, Questionnaire validation

## Abstract

**Background:**

Health Related Quality of Life (HRQOL) instruments need disease and country specific validation. In Arab countries, there is no specific validated questionnaire for assessment of HRQOL in chronic kidney disease (CKD) patients. The aim of this study was to present an Arabic translation, adaptation, and the subsequent validation of the kidney disease quality of life-short form (KDQOL-SF^TM^) version 1.3 questionnaire in a representative series of Egyptian CKD patients.

**Methods:**

KDQOL-SF^TM^ version 1.3 was translated into Arabic by two independent translators, and then subsequently translated back into English. After translation disparities were reconciled, the final Arabic questionnaire was tested by interviewing 100 pre-dialysis CKD (stage 1-4) patients randomly selected from outpatients attending the Nephrology clinic at the Main Alexandria University Hospital. Test re-test reliability was performed, with a subsample of 50 consecutive CKD patients, by two interviews 7 days apart and internal consistency estimated by Cronbach’s α. Discriminant, concept, and construct validity were assessed.

**Results:**

All items of SF-36 met the criterion for internal consistency and were reproducible. Of the 10 kidney disease targeted scales, only three had Cronbach’s α <0.7: quality of social interaction (0.23), work status (0.28), and cognitive function (0.60). All disease specific scales were reproducible. Results from discriminant validity showed that the study questionnaire could discriminate between patients’ subgroups. As for concept validity, the correlation between all domains of the questionnaire with overall health ratewas significant for all domains except for the work status, sexual function, emotional wellbeing, and role emotional. Furthermore, the correlation between the disease specific domains and the two composite summaries of SF-36 (physical and mental composite summaries) was significant for all domains except for sexual function with mental composite summary. Construct validity was indicated by the observation that the majority of the domains of the kidney disease targeted scale of KDQOL-SF^TM^ 1.3 were significantly inter-correlated. Finally, principal component analysis of the kidney disease targeted scale indicated that this part of the questionnaire could be summarized into 10 factors that together explained 70.9% of the variance.

**Conclusion:**

The results suggest that this Arabic version of the KDQOL-SF^TM^ 1.3 questionnaire is a valid and reliable tool for use in Egyptian patients with CKD.

## Background

Chronic kidney disease (CKD) is a worldwide public health problem. According to the World Health Organization, diseases of the kidney and urinary tract contribute to the global burden with approximately 850,000 deaths every year and more than 115 million disability-adjusted life years [1]. In Egypt, there is paucity of information about the incidence and prevalence of the five stages of CKD. The incidence of end stage renal disease (ESRD) increased from 65.6 pmp in 1996 to 79.9 in 2001 with gradual rise in the intervening years, while the prevalence was 225 pmp in 1996, 314 pmp in 2000, 375 pmp in 2001 and 483 pmp in 2004. Mortality among dialysis patients increased from 5.4% in 1996 to 6.2% in 2000 [2,3].

The impact of CKD on the patient’s quality of life has become increasingly recognized as an important outcome measure as patients’ perception of their well being and patient-reported outcomes (PROs) are becoming an integral part of the clinical and social evaluation of chronic illnesses and are increasingly considered a fundamental element for the assessment of the impact of therapeutic interventions. Measures of Health Related Quality of Life (HRQOL) have not only become popular investigative tools, but also have been used in an effort to define and alter models of health care delivery. The quality of life of CKD patients is a frequently overlooked yet critical dimension when evaluating the care of these patients and may offer unique information for comparing alternative treatment modalities, and for improving patient satisfaction and clinical outcomes [4].

Evaluation of HRQOL among CKD patients in Egypt can add new insight in the management of the disease as it allows the quantification of the disease consequences according to the patient’s perception and enables adjustment of medical decisions to their physical, emotional, and social needs. It also improves the adhesion to the therapeutic plan, the quality of the health care provided, and patient survival [5].

HRQOL can be assessed using generic instruments, which allow comparison between patients and general populations. Questionnaires encompassing both generic and disease-specific elements represent a valuable method of assessment which may be applied to specifically explore the impact on QOL of a range of health problems in CKD patients and test the effect of interventions in this population [6]. Hays *et al.*, (1994) [7] developed a 43 disease-specific item tool for individuals with ESRD undergoing dialysis, together with the generic Short Form36 (SF36) these items constitute the Kidney Disease Quality of Life- Short Form (KDQOL-SF^TM^) version 1.3 questionnaire. The KDQOL-SF^TM^ 1.3 has been translated into several languages including Dutch [8], Korean [9], Italian [10], Iranian [11], Brazilian [12], and Japanese [13] and has been validated for various countries. The KDQOL-SF36 questionnaire was used in Arab countries [14-16].

In the present study the KDQOL-SF^TM^ version 1.3 was modified, translated into Arabic, and culturally adapted. This Arabic version of the KDQOL-SF^TM^ 1.3 was subsequently tested among a representative sample of Egyptian pre-dialysis CKD patients to determine the psychometric properties, reliability, and validity of its dimensions for use in the assessment of HRQOL among CKD patients.

## Methods

The study protocol was approved by the Ethics Committee of the Faculty of Medicine, Alexandria University and followed International Ethical Guidelines for Epidemiological Studies [17].

### Structure of KDQOL-SF^TM^ version 1.3

The KDQOL-SF^TM^ version 1.3 combines the generic SF-36 instrument with the kidney disease specific instrument. The questionnaire consists of 80 items divided into 19 dimensions. The disease specific component of KDQOL-SF^TM^ 1.3 includes 43 kidney disease targeted items. They comprise 11 domains, including: symptom/problem list (12 items), effects of kidney disease (8 items), burden of kidney disease (4 items), cognitive function (3 items), quality of social interaction (3 items), sexual function (2 items), sleep (4 items), social support (2 items), work status (2 items), patient satisfaction (1 item), and dialysis staff encouragement (2 items). SF-36 includes 36 items that measure eight domains of functioning and wellbeing on a 100 point scale. The eight domains are: physical function (10 items), role limitations caused by physical problems (4 items), role limitations caused by emotional problems (3 items), pain (2 items), general health perceptions (5 items), social function (2 items), emotional well-being (5 items), and energy/fatigue (4 items). The final item, the overall health rate item, asks the respondents to rate their health on a 0-10 response scale. Results from the SF-36 instrument are further summarized into a physical composite summary (PCS) score and a mental composite summary (MCS) score PCS aggregates items from physical function, role physical, pain, and general health. MCS aggregates items from role emotional, emotional wellbeing, energy, and social function [18]. According to Mapes *et al*., [19], items of the kidney disease targeted scale are also summarized into kidney disease composite summary (KDCS) score on a100 point scale.

The standard scoring program of the KDQOL-SF^TM^ 1.3 is based on the Microsoft Excel 97 spreadsheet program and includes information about the computation method. The scores for each dimension range from 0 to 100, with higher scores reflecting better HRQOL. The change in health (question 2) of the SF36 scale and the 0-10 overall health rating (question 22) items are scored as single items [18].

### Translation and cultural adaptation

Permission to translate the KDQOL-SF^TM^ version 1.3 questionnaire into the Arabic language was obtained from its authors. After consulting the KDQOL working group, minimal modifications were introduced to make the questionnaire suitable for use among CKD (stage 1-4) patients. In brief, we excluded the questions about problems with access site (item 14L for hemodialysis) and catheter site (item 14M) for peritoneal dialysis, and about dialysis staff encouragement and support (items 24A and 24B). The question about satisfaction with care (item 23) was modified by changing "kidney dialysis" to "kidney disease." These modifications could potentially alter the psychometric properties of the questionnaire. Hence, there is a necessity for a new robust validation study.

The first translation step was accomplished according to the specifications established by the KDQOL working group [20]. Forward translation was done independently by the first author of this paper and by a trained bilingual translator. Both translators rated the difficulty of translating each item and the associated response choices using 0 (not at all difficult) to 100 (most difficult). One bilingual research supervisor and another Arabic translator compared the two translations and reconciled the discrepancies. The conceptual equivalency of items and response choices that were rated less than 75 were re-translated by the original translators until an acceptable independent rating of equivalence was obtained. The resulting translation was cognitively tested among 10 CKD patients. Following the cognitive testing, the KDQOL-SF^TM^ 1.3 item and response options were appropriately rewritten. The next step was to convene a panel meeting including two nephrologists, two nephrology nurses, two CKD patients and the research coordinator to evaluate the conceptual equivalence of the translation. The forward translation was then finalized based on the feedback by the panel. The Arabic version was finally back-translated into U.S. English by two additional translators following the same methodology described for the first two translators. The back translators compared their translations and came to an agreement about discrepancies. The reconciled back translation was then compared with the original U.S. English version.

### Sampling and field testing

In order to undertake field testing of the Arabic version of KDQOL-SF^TM^ 1.3 questionnaire (Additional file 1), 100 pre-dialysis CKD patients were randomly selected from patients attending the out-patient Nephrology clinic, being the pool for all nephrology patients, at the Main Alexandria University Hospital. The Main Alexandria University Hospital serves patients from different regions of the country. Patients were interviewed after their informed consent. Inclusion criteria included patients aged 18 years and above, diagnosed as pre-dialysis CKD (stage1-4). Exclusion criteria included ESRD patients, those on dialysis, had previous renal transplantation, and those with cognitive impairment. Test re-test reliability was estimated, with a subsample of 50 consecutive CKD patients, by two interviews 7 days apart.

### Floor and ceiling effects

Ceiling effects were taken as being the percentage of respondents with scores of 100 and floor effects were the percentage of respondents having a score of 0. Ceiling and floor effects should be less than 20% to ensure that the scale captures the full range of potential responses within the population, and that change over time can be detected [11].

### Psychometric evaluation of the questionnaire

Reliability: intra-class correlation coefficient (ICC) was used for assessment of the test re-test reliability, while Cronbach’s α coefficient was used to assess the internal consistency of the questionnaire [21].

Validity: discriminant validity was assessed by comparing between subgroups of the patients based on age, gender, educational level, working status, history of hypertension, history of diabetes mellitus, and stage of CKD. Concept validity was studied by analyzing the correlation coefficients between the scores of the Arabic version of the KDQOL-SF^TM^ version 1.3 and the overall health rate scale, and the correlation coefficient between the kidney disease targeted items and the scores of the two composite summaries of the SF-36. Construct validity was examined by comparing the correlation coefficients between the kidney disease targeted dimensions [22,23].

Exploratory factor analysis was used to evaluate the factor structure of the Arabic version of the KDQOL-SF^TM^ 1.3 questionnaire. Exploratory factor analysis was performed by the principal component analysis on the 39 items (after modifications) of the kidney targeted scales (after modifications) to assess the factor structure of the questionnaire [24].

### Statistical analysis

Data was summarized as median and interquartile range or as percent frequency, where appropriate. The values for the interpretation of the ICC were: <0.4, weak agreement; 0.4 to 0.75, good agreement; ≥0.75, excellent agreement. The Cronbach’s α value used as a criterion of adequate internal consistency reliability was 0.70 or higher [25].

The validity of the Arabic version of the KDQOL-SF^TM^ 1.3 was assessed by correlation analysis. Pearson’s correlation was applied to the normally distributed variables and Spearman’s rho when criteria of normality were violated. The Statistical Package for the Social Sciences (SPSS), version 16.0 for Windows was used for the analyses. The tests were two-tailed and *p* < 0.05 was considered to indicate statistical significance.

## Results

### Characteristics of the study subjects

As regards the characteristics of the 100 CKD patients included in the validation of the Arabic version of the KDQOL-SF^TM^ version 1.3 questionnaire, the median age of the study sample was 54 (42-60) years. Male patients constituted 54%. About one third of the CKD patients (34%) were university graduates and almost one fourth (24%) were illiterate. Around three fourths of the study patients (76%) were married, one fourth (25%) were current smokers, 42% were hypertensive, and 36% were diabetics. As regards the distribution of the patients by the stage of CKD, 44% of the patients were in stage 3 CKD while those in stage 4 constituted 56%.

### Forward translation

The discrepancy of forward translation between the two translators was 10%. The 10% discrepancy was resolved by the two translators by producing versions of non-concordant parts of the translations until equivalence was obtained.

### Floor effects and ceiling effects

Table 1 shows the distribution of the responses to each item of the Arabic version of KDQOL-SF^TM^ version 1.3 questionnaire. Among the kidney disease targeted scales, *work status* had the highest proportion of both ceiling and floor effects (18% and 40 %, respectively). This was followed by the *social support* item with ceiling and floor effects of 17% and 6%, respectively. *Sexual function* also had a high proportion of floor effect (16%). In the SF-36 scales, *role physical* had the highest proportion of floor effect (48%). *Role emotional* had the highest proportion of ceiling effect (50%) and a high proportion of floor effect (24%).

**Table 1:** Description and reliability of the Arabic version of KDQOL-SF^**TM **^version 1.3 among the 100 CKD patients

** Scales**	**Mean ± SD**	**Ceiling %**	**Floor %**	**ICC (n = 50)**	**Cronbach’sα(n = 100)**
**Kidney disease targeted scales**					
·**Symptoms/problems**	77.50 (11.5)	1.0	0.0	0.93	0.81
·**Effect of kidney disease**	73.84 (13.6)	2.0	0.0	0.88	0.73
·**Burden of kidney disease**	40.13 (26.6)	0.0	4.0	0.85	0.88
·**Work status**	49.00 (38.3)	18.0	40.0	0.93	0.28
·**Cognitive function**	68.73 (13.7)	0.0	0.0	0.93	0.60
·**Quality of social interaction**	71.40 (10.4)	0.0	0.0	0.92	0.23
·**Sexual function**	61.50 (23.1)	0.0	16.0	0.95	0.90
·**Sleep**	58.38 (15.9)	0.0	0.0	0.93	0.90
·**Social support**	63.17 (28.9)	17.0	6.0	0.79	0.88
·**Patient satisfaction**	65.67 (17.9)	5.0	0.0	0.94	-
**SF- 36**					
·**Physical function**	49.10 (27.3)	2.0	5.0	0.93	0.95
·**Role physical**	28.50 (32.0)	5.0	48.0	0.88	0.76
·**Pain**	44.65 (23.1)	1.0	5.0	0.94	0.87
·**General health**	37.50 (19.0)	0.0	0.0	0.92	0.83
·**Emotional well being**	60.84 (10.0)	0.0	0.0	0.89	0.70
·**Role emotional**	63.67 (41.9)	50.0	24.0	0.86	0.83
·**Social function**	54.50 (23.4)	9.0	0.0	0.79	0.88
·**Energy/Fatigue**	47.80 (14.5)	0.0	0.0	0.94	0.81
·**Overall health rate**	57.40 (8.6)	0.0	0.0	0.93	0.95

### Reliability

Intra-class correlation coefficients of the kidney disease targeted scales ranged from 0.79 to 0.95 while in the SF-36 scales ranged from 0.79 to 0.94. ICC coefficient of both *social support* in the kidney disease targeted scales and *social function* in the SF-36 scales was 0.79. In the kidney disease targeted scales, Cronbach’s α ranged from 0.23 to 0.90. Cronbach’s α for *quality of social interaction*, and *work status* was 0.23 and 0.28, respectively, while for *cognitive function*, it was 0.60. However, for the rest of kidney disease targeted scales, it was above 0.70. For the SF-36 scales, Cronbach’s α was 0.70 and above for all the items (Table 1).

### Validation

#### Discriminant validity

The Arabic version of the KDQOL-SF^TM^ version 1.3 scores were compared between subgroups of the recruited patients. Table 2 shows that females, those with history of hypertension, and those with stage 4 CKD have significantly (*p* < 0.05) lower scores for the three composite summaries (PCS, MCS, and KDCS scores). Older and non working patients scored significantly (*p* < 0.05) lower in both PCS and KDCS scores, while patients with an education less than secondary school had significantly (*p* < 0.05) lower scores for MCS and KDCS scores. Diabetic patients scored significantly lower for KDCS.

**Table 2:** Comparison of the Arabic version of KDQOL-SF version 1.3 questionnaire scores for some demographic and clinical criteria among the 100 CKD patients

**Descriptive criteria**	**PCS Mean ± (SD)**	**MCS Mean ± (SD)**	**KDCS Mean ± (SD)**
**Age**			
<50 years (35)	38.1 (10.7)	47.9 (6.4)	63.5 (13.5)
≥50 years(65)	29.9 (9.5)	45.2 (9.5)	55.3 (12.0)
*P*	<0.001	0.08	0.003
**Gender**			
Male (54)	35.0 (9.9)	49.2 (7.6)	61.3 (9.4)
Female(46)	32.4 (9.4)	41.2 (6.2)	58.9 (8.5)
*P*	0.003	0.002	0.004
**Education**			
<Secondary school (49)	32.0 (10.2)	44.6 (7.5)	54.6 (11.8)
≥Secondary school (51)	33.5 (10.7)	47.6 (6.7)	61.6 (13.5)
*p*	0.5	0.04	0.007
**Working status**			
Not working (43)	29.7 (9.7)	45.2 (7.2)	45.6 (11.5)
Working (57)	35.1 (10.8)	46.8 (7.3)	60.9 (13.7)
*p*	0.01	0.3	0.02
**History of diabetes mellitus**			
No(64)	33.7 (10.9)	46.3 (7.3)	59.1 (13.9)
Yes (36)	31.0 (10.0)	45.8 (7.5)	54.0 (11.0
*p*	0.2	0.7	0.04
**History of hypertension**			
No (58)	35.1 (10.0)	42.8 (7.1)	61.1 (10.9)
Yes(42)	32.2 (9.3)	45.8 (7.5)	57.0 (9.2)
*p*	0.001	0.005	0.01
**Stage of CKD**			
Stage 3 (44)	36.1 (10.2)	48.7 (6.3)	61.7 (9.5)
Stage 4 (56)	32.8 (9.3)	44.8 (7.3)	59.5 (8.7)
*p*	0.001	0.01	0.02

#### Concept validity

Table 3 presents the correlation between the domains of the Arabic version of the KDQOL-SF ^TM^ 1.3 and the overall health rate. Overall health rate in the kidney disease targeted scales was highly correlated with the *effect of kidney disease* (*p < 0.001*), and correlated with *symptoms/problems*, *burden of kidney disease*, *cognitive function*, *quality of social interaction*, *sleep*, and *social support* (*p* < 0.05). Overall health rate failed to correlate with *work status*, *sexual function,* and *patient satisfaction.* Among the SF-36 scales; overall health rate correlated with *physical function*, *role-physical*, *pain*, *general health*, and *social function* (*p* < 0.05). Overall health rate tended to be correlated with the energy item (*p* = 0.07) but failed to correlate with *emotional wellbeing* and *role emotional* items.

**Table 3:** Correlation between the domains and the overall health rate of the Arabic version of KDQOL-SF^**TM **^version 1.3 among the 100 CKD patients

**Scales**	**Overall health rate (r)**	**P**
**Kidney disease targeted scales**		
·**Symptoms/problems**	0.28	0.005
·**Effect of kidney disease**	0.34	<0.001
·**Burden of kidney disease**	0.31	0.003
·**Work status**	0.08	0.44
·**Cognitive function**	0.21	0.04
·**Quality of social interaction**	0.29	0.003
·**Sexual function**	0.19	0.37
·**Sleep**	0.3	0.002
·**Social support**	0.23	0.02
·**Patient satisfaction**	0.16	0.12
**SF- 36**		
·**Physical function**	0.29	0.004
·**Role physical**	0.30	0.002
·**Pain**	0.26	0.009
·**General health**	0.32	0.001
·**Emotional well being**	-0.017	0.87
·**Role emotional**	0.14	0.17
·**Social function**	0.27	0.008
·**Energy/Fatigue**	0.18	0.07

Table 4 shows the correlation between the kidney disease targeted scales and the two the main composite summaries (PCS and MCS) scores. Both PCS and MCS scores were correlated with all domains of the kidney disease targeted scales (*p* < 0.05) with the exception of MCS score which failed to correlate with the *sexual function*.

**Table 4:** Correlation between the kidney disease targeted scales and SF-36 main composite summaries of the Arabic version of KDQOL-SF^**TM **^1.3 among the 100 CKD patients

**Kidney disease targeted scales**	**PCS, r(p)**	**MCS, r(p)**
·**Symptoms/problems**	0.61 (<0.001)	0.40 (<0.001)
·**Effect of kidney disease**	0.45 (<0.001)	0.24 (0.02)
·**Burden of kidney disease**	0.68 (<0.001)	0.53 (<0.001)
·**Work status**	0.68 (<0.001)	0.37 (<0.001)
·**Cognitive functions**	0.67 (<0.001)	0.40 (<0.001)
·**Quality of social interaction**	0.38 (<0.001)	0.55 (<0.001)
·**Sexual function**	0.45 (<0.001)	0.10 (0.35)
·**Sleep**	0.61 (<0.001)	0.28 (0.006)
·**Social support**	0.24 (0.02)	0.24 (0.02)
·**Patient satisfaction**	0.41 (<0.001)	0.32 (0.001)

#### Construct validity

Most of the domains were highly correlated with correlation coefficient (r) ranging from 0.25 to 0.72 (*p* < 0.001). Other domains were weakly correlated (r: 0.20 to 0.29, *p* < 0.05). Some other domains like *sexual function* and *effects of kidney disease*, and *social support versus work status* just failed to reach statistical significance (Additional file 2).

#### Exploratory factor analysis

Factor analysis with varimax rotation revealed that the 39 kidney disease specific items encompassed the ten domains of the kidney targeted scales of the questionnaire after modifications. The Kaiser–Meyer–Olkin measure of sampling adequacy was 0.73, which is above the recommended value of 0.60, and the Bartlett’s test of sphericity was found to be highly significant (Χ^2^ = 1.830, *p* < 0.001). Moreover, all the communalities found to be above 0.5. Using these previously mentioned indicators, a factor analysis was conducted on all the 39 items; this took the form of a principal component analysis. The initial eigenvalues showed that all 39 items explained 70.9% of the variance in 10 components. Varimax rotation gave higher factor loading as compared to un-rotated factor method (factor loading ranged from 0.50 to 0.94). Social support, effect of kidney disease, and burden of kidney disease items exhibited a stronger relationship (>0.7). Low factor loadings (<0.5) were observed specially for items” Did you have difficulty concentrating or thinking?”, “Did you become confused?”, “Awaken during the night and have trouble falling asleep again?”, “Have trouble staying awake during the day?”, and “Think about the care you receive for kidney disease, in terms of your satisfaction, how would you rate the friendliness and interest shown in you as a person?”

## Discussion

This study reports the psychometric characteristics and the validation of the Arabic version of the KDQOL-SF^TM^ version 1.3 among pre-dialysis CKD patients. The study sample was considered representative of CKD patients in Egypt as both the age and gender distributions of the patients were closely similar to that found in the 9^th^ annual report of the Egyptian renal registry [2].

Differences between regions, populations, and cultures require reliability and validity assessment of measurement instruments [26]. In the Arab countries, this is the first validation study of the KDQOL-SF^TM^ version 1.3 questionnaire. Other published articles aimed at assessing the HRQOL in CKD patients. Only, one study in Saudi Arabia merely mentioned that the questionnaire (KDQOL-SF 36) was validated but there were no details [14-16].

Although the KDQOL-SF^TM^ version 1.3 questionnaire was conceived for self administration, we tested the Arabic version of the KDQOL-SF^TM^ 1.3 by interviewing 100 (stage 1-4) CKD patients. Almost one fourth (24%) of the study population were illiterate. Administrating the questionnaire by two methods, as self administration for those who could read and write, and as researcher-administered for those who were illiterate would have reduced the precision of HRQOL scores due to differences in the way of collecting information.

During the forward translation we encountered some difficulties with terms, for example; describing items that are not part of everyday life of the Egyptian people. One of these was “*Bowling or playing golf*” as these are not common sports in Egypt. Therefore, It was removed being an example given, among others, of moderate activities. Differentiating between “*Most of time*” and “*A good bit of the time*,” and between “*Quite a bit*” and “*Extremely*” posed some difficulty because most of the Egyptian people regard these responses as representing similar quantities. By the same token, “*Walking more than one mile*”, “*Walking several blocks*” or “*Walking one block*” are not usual measures of distance in Egypt. To provide a conceptually equivalent meaning of block or mile, we estimated the distance in terms of more than one tram station, one tram station or half a tram station. Translators also had difficulty in adapting “*Several flights of stairs*”, so we expressed it in the form of one floor or more than one floor.

In the field test, the distribution of responses to specific items was tested by the ceiling and floor effects. Although *work status* had the highest proportion of floor effect, this effect was smaller in our study than that reported by other studies [7-9]. This may be due to the fact that the patients included in the present study were pre-dialysis CKD patients, while those included in the other studies were dialysis patients. Also, it may be due to the younger age of the patients included in our study than other studies.

*Sexual function* also had a high percentage of floor scores, which is similar to that reported by Pakpour *et al.*, [11]. The high mean age of the patients, and the high proportion of refusal to answer this item due to the personal/sensitive nature of this question could explain the difficulties with this item in our Egyptian sample of CKD patients. On the other hand, the ceiling effect was high in the social support item which is also reported in the Netherlands study [8]. This may be explained by the closely knit nature of the Egyptian community.

For the SF-36 part of the questionnaire, *role physical* domain reported high floor effect while *role emotional* domain reported high ceiling effect. These findings were similar to those described by Cheung *et al.*, [27].

Thus floor and ceiling effects may reduce the usefulness of the above mentioned scales in this Arabic version of KDQOL-SF^TM^ 1.3.

Test re-test reliability was assessed in a sufficiently large subsample (n = 50) of the respondents and scales were found to be equal or greater than the minimal required value of 0.40. Therefore, this Arabic version of the KDQOL-SF^TM^ 1.3 provides reproducible results.

Internal consistency was acceptable for all SF-36 scales and for most of the kidney disease targeted scales. Psychometric test results were less consistent for three dimensions*, work status*, *cognitive function*, *and quality of social interaction*. For *quality of social interaction* and *work status*, Cronbach’s α was 0.23 and 0.28, respectively. These are low values compared to corresponding items in validation studies in USA [7], Japan [9], and Iran [11], but similar to those reported from the Netherlands [8]. As discussed, a possible explanation for these low values could be the complexity of the quality of social interaction item and the high percentage of non working patients (43%) in our population. Furthermore, *work status* consists of a rather limited number of items with a limited number of response categories, which could be partly responsible for the low observed results. Whatever the explanation, scores of these dimensions should be interpreted with caution in this Arabic version.

For the remaining seven dimensions of the kidney disease targeted scales and for all the dimensions of SF36, the internal consistency presented in our study was more or less similar to that observed in validation studies in other countries. The good agreement between the reliability of the scale scores of the original US version and the Arabic version confirms the measurement equivalence between them. For most of the dimensions, only small discrepancies were found, with the exception of work status and quality of social interaction, which showed lower values, compared to US reported values (Table 5). Therefore, apart from these two dimensions, results of this Arabic version provide acceptable estimates of HRQOL.

**Table 5:** Country comparison of internal consistency reliability of the KDQOL-SF^**TM **^version 1.3 questionnaire

**Scales**	**Egypt**	**US**^**(1)**^	**Japan**^**(2)**^	**Iran**^**(3)**^	**Netherland**^**(4)**^
**Kidney disease targeted scales**					
·**Symptoms/problems**	0.81	0.84	0.81	0.92	0.80
·**Effects of kidney disease**	0.73	0.82	0.79	0.89	0.76
·**Burden of kidney disease**	0.88	0.83	0.81	0.86	0.80
·**Work status**	0.28	0.83	0.69	0.71	0.39
·**Cognitive function**	0.60	0.68	0.73	0.74	0.83
·**Quality of social interaction**	0.23	0.61	0.35	0.77	0.39
·**Sexual function**	0.90	0.89	0.92	0.92	0.95
·**Sleep**	0.90	0.90	0.61	0.77	0.72
·**Social support**	0.88	0.89	0.76	0.76	0.67
**SF-36**					
·**Physical function**	0.95	0.92	0.90	0.93	….
·**Role-physical**	0.76	0.87	0.88	0.89	….
·**Pain**	0.87	0.90	0.83	0.88	….
·**General health**	0.83	0.78	0.80	0.74	….
·**Mental health**	0.70	0.80	0.84	0.73	….
·**Role-Emotional**	0.83	0.86	0.92	0.79	….
·**Social function**	0.88	0.87	0.73	0.82	….
·**Energy/Fatigue**	0.81	0.90	0.81	0.79	….

^1^ Data by Hays et al (1994).

^2^ Data by Green et al (2001).

^3^ Data by Pakpour et al (2011).

^4^ Data by Korevaar et al (2002).

Results based on the discriminant validity by subgroup comparisons showed that the Arabic version of the KDQOL-SF^TM^ version 1.3 scores could distinguish between subgroups of the patients based on age, gender, educational level and also some clinical conditions as history of hypertension diabetes mellitus, and the stage of CKD. Our results are in concordance with results from other studies in which older patients, females, those with history of comorbidities, and those with advanced stage of CKD have worse HRQOL [5,28].

Regarding concept validity, all SF-36 scales were significantly correlated with the overall health rate (*p < 0.05*), except for *role emotional* and *emotional wellbeing* domains. Moreover, for the kidney disease-specific scales, all scales were significantly inter-correlated (*p < 0.05*) with the overall health rate except for *work status*, *sexual function*, and *patient satisfaction*. Also, positive correlation coefficients were found between all the dimensions of the Arabic version of the KDQOL-SF^TM^ version 1.3 and the two composite summaries of the SF-36. The correlation coefficients between most of the dimensions of the KDQOL-SF^TM^ 1.3 were moderate to high, indicating that the instrument has an adequate construct validity.

Exploratory factor analysis of this Arabic version of the KDQOL-SF version 1.3 revealed that the kidney disease specific items could be summarized into 10 factors after the modification and removal of the domain concerned with the dialysis staff encouragement. This is supported by what was reported by Hays *et al.*, [7].

## Conclusions

Based on the findings of this study, the Arabic version of the KDQOL-SF^TM^ version 1.3 is reliable and valid. This questionnaire could be administered by the health workers to assess HRQOL among pre-dialysis CKD patients.

## Abbreviations

CKD: Chronic kidney disease; ESRD: End stage renal disease; HRQOL: Health related quality of life; PROs: Patient reported outcomes; KDQOL-SF^TM^ 1.3: Kidney Disease Quality of Life- Short Form questionnaire version 1.3; ICC: Intra class correlation; PCS: Physical composite summary; MCS: Mental composite summary, KDCS: Kidney disease composite summary.

## Competing interests

The author(s) declare that they have no competing interests

## Authors’ contributions

SA, ZMG conceptualized and designed the study. SA, SAS, NMA participated in the KDQOL-SF^TM^ 1.3 questionnaire translation. SA performed the data collection and data entry. HAS supervised the data capture. GT, SA, CT analyzed and interpreted the data. CZ, GT, SAS, SA, CT drafted and revised the manuscript critically. All authors read and approved the final manuscript.

## Pre-publication history

The pre-publication history for this paper can be accessed here:

http://www.biomedcentral.com/1471-2369/13/170/prepub

## Supplementary Material

Additional file 1 The Questionnaire: contains the Arabic version of the KDQOL-SF^**TM **^1.3 questionnaire that has been validated by the authors in this study. Click here for file

Additional file 2 A table: shows correlation between different items of kidney disease targeted scale of the Arabic version of the KDQOL-SF^**TM **^version 1.3 among the 100 CKD patients. Click here for file
