# Supplementary material for: Cultural adaptation and validation of the “Kidney Disease and Quality of Life - Short Form (KDQOL-SF™) version 1.3” questionnaire in Egypt
Source: BMC Nephrol. 2012 Dec 13;13:170. doi: 10.1186/1471-2369-13-170 (PMC3583144; doi:10.1186/1471-2369-13-170)
Supplement: Additional file 1 — The Questionnaire: contains the Arabic version of the KDQOL-SFTM 1.3 questionnaire that has been validated by the authors in this study. [file 1471-2369-13-170-S1.pdf]

رعايتك الصحيه

امراض الكلى وجودة الحياه

(KDQOL-SF™)

Version 1.3

الرقم المسلسل للمريض

RAND
1700 Main Street
Santa Monica, California 90407-2138
(310)393-0411

Kidney Disease and Quality of Life™ Short Form (KDQOL-SF™) Arabic Version 1.3 Copyright © 1993, 1994, 1995 by RAND and the University of Arizona

تمت هذه الدراسه في عام ٢٠١٢ بواسطة الدكتوره سمر عبد الحفيظ ، الدكتوره سني سلام، الدكتوره زهيره جاد، الدكتور كارمن زوكالي، الدكتوره كلوديا تورينو، الدكتور جيوفاني تريبيبي, الدكتوره هاله الوكيل, الدكتوره نهى عوض
المعهد العالي للصحه العامه- جامعة الإسكندريه البريد الإليكتروني: dr.summer8103@yahoo.ca

يخضع استخدام هذه النسخه العربيه المترجمه للحصول على أذن من المترجمين

ما هو الغرض من الدراسة؟

يجري تنفيذ هذه الدراسة بالتعاون مع الأطباء ومرضاهم. والغرض من ذلك هو تقييم جودة حياة المرضى الذين يعانون من مرض الكلى.

ماهو المطلوب مني فعله ؟

نحن نريد منك ملأ هذه الإستماره عن حالتك الصحيه اليوم ، وكيف تشعر وبعض البيانات الخاصة بك.

سرية المعلومات:

نحن لا نسأل عن إسمك ولكن سوف يتم الجمع بين إجاباتك مع اجابات المشاركين الآخرين في الإبلاغ عن النتائج التي توصلت إليها الدراسة. وسوف يتم اعتبار أي من المعلومات التي تسمح بالتعرف عليك سريه للغايه بالإضافة إلى أن كل المعلومات المجمعه سوف تستخدم فقط لغرض الدراسه ولن يتم الكشف عنها أو استخدامها لأغراض اخرى قبل الحصول على موافقتك

كيف ستكون المشاركه في الدراسه مفيده لي؟

إن المعلومات التي ستقدمها لنا ستخبرنا عن شعورك تجاه الرعايه التي تحصل عليها وأثرها على صحة المرضى. سوف تساعدنا ايضا في تقييم الرعاية الصحيه المقدمة

هل لابد لي ان اشارك ؟

انت لست مضطرا للمشاركه وملأ الإستماره كما أنه يمكنك رفض الإجابة عن أي سؤال وهذا القرار لن يؤثر على فرصتك في الحصول على الرعاية الصحية.

معلومات لملأ الإستماره:

أ- تسأل هذه الإستماره عن وجهة نظرك في صحتك و سوف تساعدنا هذه المعلومات لمعرفة كيف تشعر و مدى قدرتك على ان تقوم بأنشطتك المعتاده
ب- تحتوي هذ الإستماره على اسئله متنوعه عن صحتك وحياتك ويهمنا معرفة شعورك تجاه كلا منهم
ت- من فضلك اجب عن الأسئله برسم دائره حول الرقم الذي يدل عن الإجابه الصحيحه:

على سبيل المثال:
قد ايه كان عندك وجع في جسمك في ال ٤اسابيع اللي فاتت

مفيش وجع خالص	١
وجع بسيط	٢
وجع متوسط	٣
وجع جامد	٤
وجع جامد جدا	٥

Kidney Disease and Quality of Life™ Short Form (KDQOL-SF™) Arabic Version 1.3 Copyright © 1993, 1994, 1995 by RAND and the University of Arizona
تمت هذه الدراسه في عام ٢٠١٢ بواسطة الدكتوره سمر عبد الحفيظ ، الدكتوره سني سلام، الدكتوره زهيره جاد، الدكتور كارمن زوكالي، الدكتوره كلوديا تورينو، الدكتور جيوفاني تريبيبي, الدكتوره هاله الوكيل, الدكتوره نهى عوض
المعهد العالي للصحه العامه- جامعة الإسكندريه البريد الإليكتروني :dr.summer8103@yahoo.ca
يخضع استخدام هذه النسخه العربيه المترجمه للحصول على أذن من المترجمين

ث- توجد عدة نقاط في هذه الإستماره تسأل عن تأثير مرض الكلى على حياتك وبعضها تسأل عن المعوقات التي لها علاقه بمرضك بالكلى وآخرى عن رعايتك الصحيه .بعض الأسئلة قد تكون متشابهه ، ولكن كل واحدة مختلفة. من فضلك اجب عن كل سؤال بأمانه بقدر الإمكان. إذا لم تكن متأكدا من كيفية الإجابة على السؤال ، من فضلك ضع افضل إجابة ممكنه. هذاسوف يسمح بإعطائنا صورة دقيقة عن التجارب المختلفة للأفراد المصابين بأمراض الكلى.

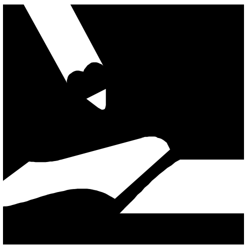

نشكرك على ملأ هذه الإستماره

Kidney Disease and Quality of Life™ Short Form (KDQOL-SF™) Arabic Version 1.3 Copyright © 1993, 1994, 1995 by RAND and the University of Arizona
تمت هذه الدراسه في عام ٢٠١٢ بواسطة الدكتوره سمر عبد الحفيظ ، الدكتوره سني سلام، الدكتوره زهيره جاد، الدكتور كارمن زوكالي، الدكتوره كلوديا تورينو، الدكتور جيوفاني تريبيبي, الدكتوره هاله الوكيل, الدكتوره نهى عوض
المعهد العالي للصحه العامه- جامعة الإسكندريه البريد الإليكتروني: dr.summer8103@yahoo.ca
يخضع استخدام هذه النسخه العربيه المترجمه للحصول على أذن من المترجمين

صحتك

<u>السؤال الأول: تقول صحتك بصفه عامة :(ارسم دائره على إجابه واحده)</u>

ممتازه	١
كويسه جدا	٢
كويسه	٣
مقبوله	٤
ضعيفه	٥

<u>السؤال الثاني: هتقدّر صحتك دلوقتي ازاي لما تقارنها بسنه فاتت؟ : (ارسم دائره على إجابه واحده)</u>

أحسن بكتير من سنه فاتت	١
أحسن شويه من سنه فاتت	٢
تقريبا زي سنه فاتت	٣
أوحش شويه من سنه فاتت	٤
أوحش بكتير من سنه فاتت	٥

Kidney Disease and Quality of Life™ Short Form (KDQOL-SF™) Arabic Version 1.3 Copyright © 1993, 1994, 1995 by RAND and the University of Arizona
تمت هذه الدراسه في عام ٢٠١٢ بواسطة الدكتوره سمر عبد الحفيظ ، الدكتوره سني سلام، الدكتوره زهيره جاد، الدكتور كارمن زوكالي، الدكتوره كلوديا تورينو، الدكتور جيوفاني تريبيبي, الدكتوره هاله الوكيل, الدكتوره نهى عوض
المعهد العالي للصحه العامه- جامعة الإسكندريه البريد الإليكتروني: dr.summer8103@yahoo.ca
يخضع استخدام هذه النسخه العربيه المترجمه للحصول على أذن من المترجمين

السؤال الثالث: النقط اللى جاية عن أنشطة أنت ممكن تعملها فى يوم عادى ... هل حالتك الصحية دلوقتي بتقلل من امكانيتك إنك تعملها ؟... وإن كان كده قد ايه ؟: (ارسم دائره على إجابه واحده من كل سطر)

	ايوه بتقللها جامد	ايوه بتقللها قليل	مش بتقللها خالص
أ- أنشطه عنيفه زي الجري أو رفع حاجات تقيله او تشارك في رياضه شديده	١	٢	٣
ب- أنشطه متوسطه ،زي نقل ترابيزه ،او الكنس	١	٢	٣
ت- شيل الطلبات من السوق	١	٢	٣
ث- طلوع كذا دور من السلالم	١	٢	٣
ج- طلوع دور واحد من السلم	١	٢	٣
ح- توطي او تركع أو تنحني	١	٢	٣
خ- تمشي كذا محطة ترام	١	٢	٣
د- تمشي محطة ترام	١	٢	٣
ذ- تمشي نص محطة ترام	١	٢	٣
ر- تستحمى او تلَبس نفسك	١	٢	٣

السؤال الرابع: في ال ٤ أسابيع اللي فاتت ،هل كان عندك أي من المشاكل اللي هنقولها في شغلك أوأي انشطه تانيه منتظمه بسبب صحة جسمك؟ (ارسم دائره على إجابه واحده من كل سطر)

	نعم	لا
أ- قللت الوقت اللي قضيته في شغلك أو اي انشطه تانيه؟	١	٢
ب- نفذت أقل من اللي كنت تحب تعمله ؟	١	٢
ت- لقيت في نوع شغل أو أنشطه معينه مقدرتش تعملها؟	١	٢
ث- كان في صعوبه انك تعمل الشغل أوأي انشطه تانيه(مثلا يحتاج لمجهود زياده)	١	٢

السؤال الخامس: في ال ٤ أسابيع اللي فاتت ،هل كان عندك أي من المشاكل اللي هنقولها في شغلك أوأي انشطه تانيه يوميه منتظمه نتيجه لأي مشاكل عاطفيه (زي الإحساس بالإكتئاب أو القلق)؟ (ارسم دائره على إجابه واحده من كل سطر)

	نعم	لا
أ- قللت الوقت اللي قضيته في شغلك أو اي انشطه تانيه؟	١	٢
ب- نفذت أقل من اللي كنت تحب تعمله ؟	١	٢
ت- معملتش الشغل أوي أنشطه تانيه بعنايه زي العاده؟	١	٢

السؤال السادس: في ال ٤ الاسابيع اللي فاتت صحة جسمك أو مشاكلك العاطفيه أثرت على أنشطتك الإجتماعيه العاديه مع عيلتك واصحابك وجيرانك قد ايه؟ (ارسم دائره على إجابه واحده)

مفيش تأثير خالص	١
أثرت بسيط	٢
أثرت متوسط	٣
أثرت جامد	٤
أثرت جامد جدا	٥

السؤال السابع: قد ايه كان عندك وجع في جسمك فى ال٤ اسابيع اللى فاتت؟ (ارسم دائره على إجابه واحده)

مفيش وجع خالص	١
وجع بسيط جدا	٢
وجع بسيط	٣
وجع متوسط	٤
وجع جامد	٥
وجع جامد جدا	٦

Kidney Disease and Quality of Life™ Short Form (KDQOL-SF™) Arabic Version 1.3 Copyright © 1993, 1994, 1995 by RAND and the University of Arizona
تمت هذه الدراسه في عام ٢٠١٢ بواسطة الدكتوره سمر عبد الحفيظ ، الدكتوره سني سلام، الدكتوره زهيره جاد، الدكتور كارمن زوكالي، الدكتوره كلوديا تورينو، الدكتور جيوفاني تريبيبي, الدكتوره هاله الوكيل, الدكتوره نهى عوض
المعهد العالي للصحه العامه- جامعة الإسكندريه البريد الإليكتروني :dr.summer8103@yahoo.ca
يخضع استخدام هذه النسخه العربيه المترجمه للحصول على أذن من المترجمين

السؤال الثامن: في ال٤ أسابيع اللي فاتت قد ايه الألم منعك من شغلك العادى (جوه وبره البيت)؟(ارسم دائره على إجابه واحده)

ماحصلش خالص	١
حاجه بسيطه	٢
متوسط	٣
كتير	٤
كتير جدا	٥

Kidney Disease and Quality of Life™ Short Form (KDQOL-SF™) Arabic Version 1.3 Copyright © 1993, 1994, 1995 by RAND and the University of Arizona
تمت هذه الدراسه في عام ٢٠١٢ بواسطة الدكتوره سمر عبد الحفيظ ، الدكتوره سني سلام، الدكتوره زهيره جاد، الدكتور كارمن زوكالي، الدكتوره كلوديا تورينو، الدكتور جيوفاني تريبيبي, الدكتوره هاله الوكيل, الدكتوره نهى عوض
المعهد العالي للصحه العامه- جامعة الإسكندريه البريد الإليكتروني :dr.summer8103@yahoo.ca
يخضع استخدام هذه النسخه العربيه المترجمه للحصول على أذن من المترجمين

السؤال التاسع: الأسئله دى عن إحساسك والأمور كانت ماشيه ازاى معاك في ال٤ اسابيع اللي فاتت. لكل سؤال اختار الإجابه الواحده اللى كانت أقرب إلى احساسك:
قد ايه من الوقت في ال٤ أسابيع اللى فاتت.... (ارسم دائره على إجابه واحده من كل سطر)

	طول الوقت	معظم الوقت	وقت كتير	احيانا	وقت قليل	ما حصلش ابدا
أ- حسيت إنك مليان حيويه؟	١	٢	٣	٤	٥	٦
ب- كنت شخص عصبي ومتوتر جدا؟	١	٢	٣	٤	٥	٦
ت- حسيت انك متضايق لدرجه إن مفيش حاجه ممكن تبسطك؟	١	٢	٣	٤	٥	٦
ث- كنت حاسس بالهدوء والأمان؟	١	٢	٣	٤	٥	٦
ج- كنت حاسس إن عندك طاقه كتيره؟	١	٢	٣	٤	٥	٦
ح- كنت حاسس إنك مقبوض وحزين؟	١	٢	٣	٤	٥	٦
خ- حسيت إنك مهدود	١	٢	٣	٤	٥	٦
د- كنت حاسس إنك شخص سعيد	١	٢	٣	٤	٥	٦
ذ- حسيت إنك تعبان	١	٢	٣	٤	٥	٦

Kidney Disease and Quality of Life™ Short Form (KDQOL-SF™) Arabic Version 1.3 Copyright © 1993, 1994, 1995 by RAND and the University of Arizona
تمت هذه الدراسه في عام ٢٠١٢ بواسطة الدكتوره سمر عبد الحفيظ ، الدكتوره سني سلام، الدكتوره زهيره جاد، الدكتور كارمن زوكالي، الدكتوره كلوديا تورينو، الدكتور جيوفاني تريبيبي, الدكتوره هاله الوكيل, الدكتوره نهى عوض
المعهد العالي للصحه العامه- جامعة الإسكندريه البريد الإليكتروني: dr.summer8103@yahoo.ca
يخضع استخدام هذه النسخه العربيه المترجمه للحصول على أذن من المترجمين

السؤال العاشر: في ال٤ اسابيع اللي فاتت قد ايه من الوقت صحة جسمك أو مشاكلك العاطفيه أثرت على انشطتك الإجتماعيه(زي زيارة الأصحاب والأقارب)؟ (ارسم دائره على إجابه واحده)

طول الوقت	١
معظم الوقت	٢
احيانا	٣
وقت قليل	٤
ماحصلش ابدا	٥

السؤال الحادي عشر: اختر الإجابه اللي بتوصف حالتك اكتر.(ارسم دائره على إجابه واحده من كل سطر)

	اكيد حقيقي	غالبا حقيقي	مش عارف	غالبا غلط	اكيدغلط
أ- باين إني بعيي بسهوله اكتر من الناس التانيه	١	٢	٣	٤	٥
ب- صحتي كويسه زي اي حد اعرفه	١	٢	٣	٤	٥
ت- متوقع ان صحتي هتتدهور	١	٢	٣	٤	٥
ث- صحتي ممتازه	١	٢	٣	٤	٥

Kidney Disease and Quality of Life™ Short Form (KDQOL-SF™) Arabic Version 1.3 Copyright © 1993, 1994, 1995 by RAND and the University of Arizona
تمت هذه الدراسه في عام ٢٠١٢ بواسطة الدكتوره سمر عبد الحفيظ ، الدكتوره سني سلام، الدكتوره زهيره جاد، الدكتور كارمن زوكالي، الدكتوره كلوديا تورينو، الدكتور جيوفاني تريبيبي, الدكتوره هاله الوكيل, الدكتوره نهى عوض
المعهد العالي للصحه العامه- جامعة الإسكندريه البريد الإليكتروني: dr.summer8103@yahoo.ca
يخضع استخدام هذه النسخه العربيه المترجمه للحصول على أذن من المترجمين

مرضك بالكلى

<u>السؤال الثاني عشر: قولنا قد ايه الكلام اللي جاي صح او غلط بالنسبه لك؟ (ارسم دائره على إجابه واحده من كل سطر)</u>

	اكيد حقيقي	غالبا حقيقي	مش عارف	غالبا غلط	اكيد غلط
أ- مرض الكلى اللى عندى بياثر زياده عن اللزوم على حياتى	١	٢	٣	٤	٥
ب- زياده عن اللزوم من وقتي بيضيع بسبب مرضي بالكلى	١	٢	٣	٤	٥
ت- باحس اني زهقان بسبب مرضي بالكلى	١	٢	٣	٤	٥
ث- باحس اني حمل على أسرتي	١	٢	٣	٤	٥

Kidney Disease and Quality of Life™ Short Form (KDQOL-SF™) Arabic Version 1.3 Copyright © 1993, 1994, 1995 by RAND and the University of Arizona
تمت هذه الدراسه في عام ٢٠١٢ بواسطة الدكتوره سمر عبد الحفيظ ، الدكتوره سني سلام، الدكتوره زهيره جاد، الدكتور كارمن زوكالي، الدكتوره كلوديا تورينو، الدكتور جيوفاني تريبيبي, الدكتوره هاله الوكيل, الدكتوره نهى عوض
المعهد العالي للصحه العامه- جامعة الإسكندريه البريد الإليكتروني :dr.summer8103@yahoo.ca
يخضع استخدام هذه النسخه العربيه المترجمه للحصول على أذن من المترجمين

السؤال الثالث عشر: : الأسئله دى عن إحساسك والأمور كانت ماشيه ازاى معاك في ال٤ اسابيع اللي فاتت. لكل سؤال اختار الإجابه الواحده اللي كانت أقرب إلى احساسك:
قد ايه من الوقت في ال٤ أسابيع اللي فاتت.........(ارسم دائره على إجابه واحده من كل سطر)

	ماحصلش ابدا	وقت قليل	احيانا	وقت كتير	معظم الوقت	طول الوقت
أ- عزلت نفسك عن الناس اللي حواليك	١	٢	٣	٤	٥	٦
ب- كان رد فعلك بطئ لحاجات اتقالت او اتعملت	١	٢	٣	٤	٥	٦
ت- اتصرفت بتوتر مع الناس اللي حواليك	١	٢	٣	٤	٥	٦
ث- كان عندك صعوبه في التركيز او التفكير	١	٢	٣	٤	٥	٦
ج- علاقتك كانت كويسه مع الناس	١	٢	٣	٤	٥	٦
ح- كنت حاسس انك متلخبط	١	٢	٣	٤	٥	٦

Kidney Disease and Quality of Life™ Short Form (KDQOL-SF™) Arabic Version 1.3 Copyright © 1993, 1994, 1995 by RAND and the University of Arizona
تمت هذه الدراسه في عام ٢٠١٢ بواسطة الدكتوره سمر عبد الحفيظ ، الدكتوره سني سلام، الدكتوره زهيره جاد، الدكتور كارمن زوكالي، الدكتوره كلوديا تورينو، الدكتور جيوفاني تريبيبي, الدكتوره هاله الوكيل, الدكتوره نهى عوض
المعهد العالي للصحه العامه- جامعة الإسكندريه البريد الإليكتروني :dr.summer8103@yahoo.ca
يخضع استخدام هذه النسخه العربيه المترجمه للحصول على أذن من المترجمين

السؤال الرابع عشر: لأي درجه كنت متضايق في ال٤ اسابيع اللي فاتت من اللي جاي؟

(ارسم دائره على إجابه واحده من كل سطر)

	ماكنتش متضايق خالص	كنت متضايق شويه	كنت متضايق بدرجه متوسطه	كنت متضايق كتير	كنت متضايق كتير جدا
أ- حرقان في عضلاتك	١	٢	٣	٤	٥
ب- وجع في الصدر	١	٢	٣	٤	٥
ت- شد عضلي	١	٢	٣	٤	٥
ث- اكلان في جلدك	١	٢	٣	٤	٥
ج- جلدك ناشف	١	٢	٣	٤	٥
ح- كرشة نفس	١	٢	٣	٤	٥
خ- كنت حاسس هيغمى عليك أو دوغه	١	٢	٣	٤	٥
د- ملكش نفس للأكل	١	٢	٣	٤	٥
ذ- مهدود وهبطان	١	٢	٣	٤	٥
ر- تنميل في ايديك أو رجلك	١	٢	٣	٤	٥
ز- غممان نفس او تعب في المعده	١	٢	٣	٤	٥

Kidney Disease and Quality of Life™ Short Form (KDQOL-SF™) Arabic Version 1.3 Copyright © 1993, 1994, 1995 by RAND and the University of Arizona
تمت هذه الدراسه في عام ٢٠١٢ بواسطة الدكتوره سمر عبد الحفيظ ، الدكتوره سني سلام، الدكتوره زهيره جاد، الدكتور كارمن زوكالي، الدكتوره كلوديا تورينو، الدكتور جيوفاني تريبيبي, الدكتوره هاله الوكيل, الدكتوره نهى عوض
المعهد العالي للصحه العامه- جامعة الإسكندريه البريد الإليكتروني :dr.summer8103@yahoo.ca
يخضع استخدام هذه النسخه العربيه المترجمه للحصول على أذن من المترجمين

تأثير مرض الكلى على حياتك اليوميه

السؤال الخامس عشر: بعض الناس بتتضايق من تأثير مرض الكلى على حياتهم اليوميه والبعض لا. قد ايه مرض الكلى بيضايقك بخصوص الكلام اللي جاي؟(ارسم دائره على إجابه واحده من كل سطر)

	ماكنتش متضايق خالص	كنت متضايق شويه	كنت متضايق بدرجه متوسطه	كنت متضايق كتير	كنت متضايق كتير جدا
أ- تقليل كمية سوائل	١	٢	٣	٤	٥
ب- تقليل كمية الأكل	١	٢	٣	٤	٥
ت- مقدرتك على الشغل في البيت	١	٢	٣	٤	٥
ث- مقدرتك على السفر	١	٢	٣	٤	٥
ج- معتمد على الدكاتره	١	٢	٣	٤	٥
ح- قلقان وتعبان بسبب مرضك بالكلى	١	٢	٣	٤	٥
خ- حياتك الجنسيه	١	٢	٣	٤	٥
د- شكلك الشخصي	١	٢	٣	٤	٥

Kidney Disease and Quality of Life™ Short Form (KDQOL-SF™) Arabic Version 1.3 Copyright © 1993, 1994, 1995 by RAND and the University of Arizona
تمت هذه الدراسه في عام ٢٠١٢ بواسطة الدكتوره سمر عبد الحفيظ ، الدكتوره سني سلام، الدكتوره زهيره جاد، الدكتور كارمن زوكالي، الدكتوره كلوديا تورينو، الدكتور جيوفاني تريبيبي, الدكتوره هاله الوكيل, الدكتوره نهى عوض
المعهد العالي للصحه العامه- جامعة الإسكندريه البريد الإليكتروني :dr.summer8103@yahoo.ca
يخضع استخدام هذه النسخه العربيه المترجمه للحصول على أذن من المترجمين

<u>الأسئلة الثلاثة اللي جايه شخصيه ولها علاقه بالنشاط الجنسي ، ولكن إجاباتك مهمه لفهم مرض الكلى بيأثر ازاي على حياة الناس .</u>

<u>السؤال السادس عشر: هل كان عندك اي نشاط جنسي في ال٤ أسابيع اللي فاتت؟</u>
<u>(ارسم دائره على إجابه واحده)</u>

لا (١) اذا كانت الإجابه لا اذهب الى السؤال ١٧
نعم (٢)

↓

قد ايه كان عندك مشكله في ال٤ اسابيع اللي فاتت في النقط اللي جايه: (ارسم دائره على إجابه واحده من كل سطر)

	مفيش مشكله خالص	مشكله قليله	في مشكله شويه	مشكله جامده	مشكله جامده جدا
أ- مبسوط	١	٢	٣	٤	٥
ب- في إثاره جنسيه	١	٢	٣	٤	٥

في السؤال اللي جاي قولنا تقديرك لمعدل نومك باستخدام مقياس مابين ٠ يمثل "وحش جدا" الى ١٠ "كويس جدا ". لو بتحس أن نومك بين "وحش جدا" و "كويس جدا" ، لو سمحت ارسم دائره على رقم ٥. إذا كنت بتحس أن نومك احسن من المستوى ٥ بدرجه ارسم دائره على ٦. إذا كنت بتحس ان نومك أوحش من مستوى ٥ بدرجه، ارسم دائره على ٤ (وهكذا)

<u>السؤال السابع عشر:</u> على المقياس من ١ الى ١٠ بتقدر نومك (ارسم دائره على إجابه واحده)

٠	١	٢	٣	٤	٥	٦	٧	٨	٩	١٠
وحش جدا										كويس جدا

Kidney Disease and Quality of Life™ Short Form (KDQOL-SF™) Arabic Version 1.3 Copyright © 1993, 1994, 1995 by RAND and the University of Arizona
تمت هذه الدراسه في عام ٢٠١٢ بواسطة الدكتوره سمر عبد الحفيظ ، الدكتوره سني سلام، الدكتوره زهيره جاد، الدكتور كارمن زوكالي، الدكتوره كلوديا تورينو، الدكتور جيوفاني تريبيبي, الدكتوره هاله الوكيل, الدكتوره نهى عوض
المعهد العالي للصحه العامه- جامعة الإسكندريه البريد الإليكتروني :dr.summer8103@yahoo.ca
يخضع استخدام هذه النسخه العربيه المترجمه للحصول على أذن من المترجمين

السؤال الثامن عشر: في ال٤ اسابيع اللي فاتت قد ايه أنت: (ارسم دائره على إجابه واحده من كل سطر)

	ماحصلش ابدا	وقت قليل	احيانا	وقت كتير	معظم الوقت	طول الوقت
أ- صحيت بالليل وكان صعب تنام تاني	١	٢	٣	٤	٥	٦
ب- نمت الكميه اللي أنت محتاجها	١	٢	٣	٤	٥	٦
ت- كان عندك مشكله انك تفضل صاحي طول النهار	١	٢	٣	٤	٥	٦

السؤال التاسع عشر: بخصوص أهلك واصحابك أنت قد ايه راض عن: (ارسم دائره على إجابه واحده)

	مش راض جدا	مش راض شويه	راض شويه	راض جدا
أ- كمية الوقت اللي بتقدر تقضيه مع اهلك واصحابك	١	٢	٣	٤
ب- مساندة أهلك واصحابك	١	٢	٣	٤

Kidney Disease and Quality of Life™ Short Form (KDQOL-SF™) Arabic Version 1.3 Copyright © 1993, 1994, 1995 by RAND and the University of Arizona
تمت هذه الدراسه في عام ٢٠١٢ بواسطة الدكتوره سمر عبد الحفيظ ، الدكتوره سني سلام، الدكتوره زهيره جاد، الدكتور كارمن زوكالي، الدكتوره كلوديا تورينو، الدكتور جيوفاني تريبيبي, الدكتوره هاله الوكيل, الدكتوره نهى عوض
المعهد العالي للصحه العامه- جامعة الإسكندريه البريد الإليكتروني: dr.summer8103@yahoo.ca
يخضع استخدام هذه النسخه العربيه المترجمه للحصول على أذن من المترجمين

السؤال العشرون: في ال٤ اسابيع اللي فاتت هل كنت بتشتغل في وظيفه بأجر؟ (ارسم دائره على إجابه واحده)

(١) نعم (٢) لا

السؤال الواحد والعشرون: هل حالتك الصحيه بتمنعك إنك تشتغل بأجر؟ (ارسم دائره على إجابه واحده)

(١) نعم (٢) لا

السؤال الثاني والعشرون: بصفه عامه بتقدر صحتك ازاي؟ (ارسم دائره على إجابه واحده)

٠	١	٢	٣	٤	٥	٦	٧	٨	٩	١٠
اسوأ مايمكن (لدرجة الموت أو أكثر)					نص المسافه بين الأسوأ والأحسن					صحه من أحسن مايمكن

Kidney Disease and Quality of Life™ Short Form (KDQOL-SF™) Arabic Version 1.3 Copyright © 1993, 1994, 1995 by RAND and the University of Arizona
تمت هذه الدراسه في عام ٢٠١٢ بواسطة الدكتوره سمر عبد الحفيظ ، الدكتوره سني سلام، الدكتوره زهيره جاد، الدكتور كارمن زوكالي، الدكتوره كلوديا تورينو، الدكتور جيوفاني تريبيبي, الدكتوره هاله الوكيل, الدكتوره نهى عوض
المعهد العالي للصحه العامه- جامعة الإسكندريه البريد الإليكتروني: dr.summer8103@yahoo.ca
يخضع استخدام هذه النسخه العربيه المترجمه للحصول على أذن من المترجمين

الرضا عن الرعايه

السؤال الثالث والعشرون :فكر في الرعايه الصحيه اللي بتاخدها لمرض الكلى. من ناحية رضاك عن الخدمه ايه تقييمك عن معاملتك بلطف والإهتمام بيك كشخص: (ارسم دائره على إجابه واحده)

ضعيفه جدا	١
ضعيف	٢
مقبوله	٣
كويسه	٤
كويسه جدا	٥
ممتازه	٦
اكتر من ممتاز	٧

السؤال الرابع والعشرون: لاغي الفقره أ و ب

Kidney Disease and Quality of Life™ Short Form (KDQOL-SF™) Arabic Version 1.3 Copyright © 1993, 1994, 1995 by RAND and the University of Arizona
تمت هذه الدراسه في عام ٢٠١٢ بواسطة الدكتوره سمر عبد الحفيظ ، الدكتوره سني سلام، الدكتوره زهيره جاد، الدكتور كارمن زوكالي، الدكتوره كلوديا تورينو، الدكتور جيوفاني تريبيبي, الدكتوره هاله الوكيل, الدكتوره نهى عوض
المعهد العالي للصحه العامه- جامعة الإسكندريه البريد الإليكتروني :dr.summer8103@yahoo.ca
يخضع استخدام هذه النسخه العربيه المترجمه للحصول على أذن من المترجمين
