# Supplementary material for: Cultural adaptation and validation of the “Kidney Disease and Quality of Life - Short Form (KDQOL-SF™) version 1.3” questionnaire in Egypt
Source: BMC Nephrol. 2012 Dec 13;13:170. doi: 10.1186/1471-2369-13-170 (PMC3583144; doi:10.1186/1471-2369-13-170)
Supplement: Additional file 2 — A table: shows correlation between different items of kidney disease targeted scale of the Arabic version of the KDQOL-SFTM version 1.3 among the 100 CKD patients. [file 1471-2369-13-170-S2.docx]

**Appendix: Correlation between different items of kidney disease targeted scale of the Arabic version of the KDQOL-SF^TM^ version 1.3 among the 100 CKD patients**

| **S10** | **S9** | **S8** | **S7** | **S6** | **S5** | **S4** | **S3** | **S2** | **S1** | **Kidney disease targeted scale,**  **r(p)** |
| --- | --- | --- | --- | --- | --- | --- | --- | --- | --- | --- |
|  |  |  |  |  |  |  |  |  |  | **Symptoms/ problems (S1)** |
|  |  |  |  |  |  |  |  |  | 0.59(<0.001)^®^ | **Effects of kidney disease (S2)** |
|  |  |  |  |  |  |  |  | 0.54(<0.001)^®^ | 0.67(<0.001)^®^ | **Burden of kidney disease (S3)** |
|  |  |  |  |  |  |  | 0.59(<0.001)^®^ | 0.36(<0.001)^®^ | 0.55(<0.001)^®^ | **Work status(S4)** |
|  |  |  |  |  |  | 0.60(<0.001)^®^ | 0.67(<0.001)^®^ | 0.29(0.004)^*^ | 0.72(<0.001)^®^ | **Cognitive functions(S5)** |
|  |  |  |  |  | 0.53(<0.001)^®^ | 0.45(<0.001)^®^ | 0.47(<0.001)^®^ | 0.27(0.006)^*^ | 0.53(<0.001)^®^ | **Quality of social interaction(S6)** |
|  |  |  |  | 0.25(<0.001)^®^ | 0.37(<0.001)^®^ | 0.46(<0.001)^®^ | 0.35(<0.001)^®^ | 0.19(0.06) | 0.33(<0.001)^®^ | **Sexual function**  **(S7)** |
|  |  |  | 0.36(<0.001)^®^ | 0.39(<0.001)^®^ | 0.43(<0.001)^®^ | 0.51(<0.001)^®^ | 0.58(<0.001)^®^ | 0.53(<0.001)^®^ | 0.61(<0.001)^®^ | **Sleep (S8)** |
|  |  | 0.23(0.02)^*^ | 0.09(0.36) | 0.35(<0.001)^®^ | 0.20(0.04)^*^ | 0.18(0.08) | 0.33(0.001)^*^ | 0.36(<0.001)^®^ | 0.39(<0.001)^®^ | **Social support (S9)** |
|  | 0.29(0.004)^*^ | 0.33(<0.001)^®^ | 0.19(0.60) | 0.26(<0.001)^®^ | 0.52(<0.001)^®^ | 0.44(<0.001)^®^ | 0.50(<0.001)^®^ | 0.29(0.003)^*^ | 0.50(<0.001)^®^ | **Patient satisfaction (S10)** |

^®^ Significant p<0.001

^*^ Significant p<0.05
